# Exploring the perspectives and practices of humanitarian actors towards the Participation Revolution in humanitarian digital health responses: a qualitative study

**DOI:** 10.1186/s12992-024-01042-y

**Published:** 2024-04-26

**Authors:** Jennifer Benson, Meret Lakeberg, Tilman Brand

**Affiliations:** 1 Health Sciences Bremen, University of Bremen, Bremen, Germany; 2 Department Prevention and Evaluation, Leibniz Institute for Prevention Research and Epidemiology – BIPS, Bremen, Germany; 3 Leibniz Science Campus Digital Public Health, Bremen, Germany

**Keywords:** Digital health, Humanitarian, Health, Inequity, LMIC, Localisation, Participation, Power

## Abstract

**Background:**

As crises escalate worldwide, there is an increasing demand for innovative solutions to enhance humanitarian outcomes. Within this landscape, digital health tools have emerged as promising solutions to tackle certain health challenges. The integration of digital health tools within the international humanitarian system provides an opportunity to reflect upon the system’s paternalistic tendencies, driven largely by Global North organisations, that perpetuate existing inequities in the Global South, where the majority of crises occur. *The Participation Revolution*, a fundamental pillar of *the Localisation Agenda*, seeks to address these inequities by advocating for greater participation from crisis-affected people in response efforts. Despite being widely accepted as a best practice; a gap remains between the rhetoric and practice of participation in humanitarian response efforts. This study explores the extent and nature of participatory action within contemporary humanitarian digital health projects, highlighting participatory barriers and tensions and offering potential solutions to bridge the participation gap to enhance transformative change in humanitarian response efforts.

**Methods:**

Sixteen qualitative interviews were conducted with humanitarian health practitioners and experts to retrospectively explored participatory practices within their digital health projects. The interviews were structured and analysed according to the Localisation Performance Measurement Framework’s participation indicators and thematically, following the Framework Method. The study was guided by the COREQ checklist for quality reporting.

**Results:**

Varied participatory formats, including focus groups and interviews, demonstrated modest progress towards participation indicators. However, the extent of influence and power held by crisis-affected people during participation remained limited in terms of breadth and depth. Participatory barriers emerged under four key themes: project processes, health evidence, technology infrastructure and the crisis context. Lessons for leveraging participatory digital health humanitarian interventions were conducting thorough pre-project assessments and maintaining engagement with crisis-affected populations throughout and after humanitarian action.

**Conclusion:**

The emerging barriers were instrumental in shaping the limited participatory reality and have implications: Failing to engage crisis-affected people risks perpetuating inequalities and causing harm. To advance *the Participation Revolution* for humanitarian digital health response efforts, the major participatory barriers should be addressed to improve humanitarian efficiency and digital health efficacy and uphold the rights of crisis-affected people.

**Supplementary Information:**

The online version contains supplementary material available at 10.1186/s12992-024-01042-y.

## Background

As humanitarian crises continue to escalate [1–3], on a global scale [4], there is an urgent demand for innovative approaches to improve humanitarian outcomes. In today’s increasingly digitalised world, technology offers a broad potential to respond to this challenge. Within the humanitarian health arena, digital health tools refer to diverse digital solutions that leverage technology to enhance healthcare delivery, access, and outcomes for crisis-affected people (CAP). These technologies can include but are not limited to mobile apps, telehealth platforms, electronic health records, wearable devices, remote monitoring systems, and communication tools. The WHO supports the integration of such digital interventions in the pursuit of universal health coverage and health systems strengthening [5].

Digital health tools can prove valuable in a number of humanitarian crisis contexts. Examples include disasters with damaged healthcare infrastructure, conflict zones with disrupted healthcare access, disease outbreaks with curtailed movement, or displacement settlements away from support structures [6]. In these scenarios, digital health tools can enable remote consultations, patient management, outbreak surveillance as well as health promotion and disease prevention communications. This can be delivered freely, around the clock, in a number of formats, directly to those who need it, all without a physical presence. Across these situations, digital health tools offer promising solutions to bridge healthcare gaps, enhance communication between providers and CAP, and ultimately improve healthcare delivery in resource-scarce and challenging environments. Whilst these aspects assert that digital health tools are inherently a positive and benevolent force, it is crucial to recognise the nuanced implications of these statements. The convenience of digital tools carries with them often unseen paternalistic undertones. For example, offering ‘free of charge’ digital health services may go along with expectations of gratitude and conceal how user data becomes the currency of using digital health tools [7, 8]. This leads to ethical considerations such as safeguarding data and navigating language and literacy barriers or power differentials which may impact an individual’s participation understanding or agreement. Providing local communities with an active role in both the development and delivery of digital services could overcome such paternalistic tendencies, adding to their digital empowerment whilst improving health outcomes.

However, introducing digital tools into crisis contexts is not a humanitarian response panacea. Recognition of this reality is crucial when considering pre-existing digital divides that persist even in high-income countries. In such contexts, digital exclusion prevents certain segments of the population from fully benefiting from technological advancements [9, 10]. This exclusionary dynamic is likely to be exacerbated in crisis settings where economic instability, limited access to hardware and constrained support for utilising digital tools become pronounced challenges. This acknowledgement prompts critical reflection on the potential pitfalls associated with the widespread introduction of digital technologies in the context of humanitarian crises. Increased reliance upon digital solutions may exclude vulnerable populations, further marginalising those already disproportionality affected by crises. Thus, a nuanced and context-specific approach is essential to mitigate the risks associated with digital interventions to ensure that technologies contribute towards a more equitable, ethical and inclusive humanitarian response.

Whilst crisis prevalence sits within the Global South, the humanitarian system is primarily driven by Global North organisations [11–13]. As such, response efforts tend to be paternalistic [14, 15] and perpetuate the very structural inequalities they aim to address, deepening power imbalances and failing to reach those most in need [16, 17]. Recognising the imperative for transformative change within the humanitarian system, *the Localisation Agenda* (2016) was introduced [18]. A central tenet of this Agenda is *the Participation Revolution* [19, 20], enshrining the fundamental right of CAP to participate in decision-making that affects them [21], to facilitate an effective humanitarian response. *The Participation Revolution* represents a paradigm shift away from the conception of inactive beneficiaries and towards CAP as empowered, active change agents. While participatory action in general gains traction, key guidelines, such as WHO’s Recommendations on Digital Interventions for Health Systems Strengthening [5], do not extend this far. The importance of digital acceptability according to context and culture is outlined, however the processes behind achieving this are not explored.

In the humanitarian sphere, community engagement and participatory activities are approaches that can bring response organisations and CAP together in designing and delivering response activities. Arnstein’s model of Citizen Participation categorises participatory events according to the influence and the overall power that citizens hold within them. The model ranges from the lowest levels with no participation, to the mid-levels, with a tokenistic level of participation and minor influence, and the top, ideal level, being citizen control and empowerment [22]. This model can be incorporated into humanitarian response design, integrating meaningful participation to increase legitimacy and accountability, gain community trust, foster a sense of ownership, and reinforce the ethical principles of respect and dignity.

Local cultures and contexts play a pivotal role in health interventions: For example, socio-cultural factors can profoundly influence health behaviours and epidemiological outcomes. This necessitates the incorporation of unique cultural and context-specific aspects within response efforts to successfully address health challenges [23]. Participatory and collaborative approaches can bridge cultural and contextual divides, enabling CAP to have a role in tackling their own healthcare challenges and fostering not only effective interventions but also ethical and culturally sensitive responses.

Various guidelines, including the Red Cross Code of Conduct (1992) [24], the Humanitarian Charter (2000) within the Sphere Standards [25], the Good Donorship Principles (2003) [26] and the Interagency Standing Committee’s (IASC) Commitments For Accountability To Affected People [27] all emphasise the importance of meaningful participation to achieve transformative change. In today’s increasingly digitalised world, the shift towards localisation and participation is gaining traction in both humanitarian and digital health spheres. Technology offers a unique opportunity to foster greater collaboration with CAP in response to efforts, contributing towards *the Participation Revolution*.

Meaningful participatory activities, challenging knowledge hierarchies and incorporating greater reflexivity are approaches that can enable redistribution of power from providers to communities, promoting local ownership and decision-making. For instance, Lokot and Wake [28, 29] found that collaborative co-production methodologies between humanitarian actors and CAP can further enhance stakeholder buy-in, foster two-way capacity development and establish long-term partnerships [12, 30]. Capacity development in this respect extends beyond mere technical skills. Firstly, it involves empowering communities to actively participate, assert and demand their rights, and engage in decision-making processes affecting them. Secondly, it entails enhancing the implementing agencies’ appreciation of the context, culture, and recognition of influencing factors within the implementation environment. By considering communities as experts in their own rights and leaders of locally driven health solutions, this approach can address power imbalances and promote more effective and sustainable outcomes. These approaches align with Robehmed [31] and Moore [32] in moving away from traditional top-down approaches, fostering more inclusive and responsive humanitarian action [14].

Putting these approaches into practice, Greenhalgh et al. [33] outlined numerous frameworks and guidelines for participatory action and local adaptation. Alongside this, they highlighted how these can maximise project benefits, empower and protect vulnerable groups, and build intervention legitimacy [33, 34]. Similar frameworks have been successfully implemented in various humanitarian settings, as identified by Rass [35] and Joseph [36] who found that such participatory approaches led to more sustainable and impactful interventions.

One such framework, the Localisation Performance Measurement Framework (LPMF) [37] captures active CAP participation within two qualitative indicators (section six), encompassing the involvement of CAP in assessing needs, prioritising assistance, identifying recipients, providing feedback and informing key policies and standards [37]. Such frameworks contribute towards capturing and measuring progress towards transformation change in humanitarian interventions.

Despite the growing literature, a recent qualitative study [38] revealed a paradoxical humanitarian reality where practitioners recognise the importance and benefits of involving CAP in humanitarian action whilst simultaneously failing to involve them in any meaningful manner. A similar dearth of participatory action was identified within a recent scoping study [39] that sought to discover and critically analyse participatory action in digital health interventions. These findings were consistent with other related digital health and humanitarian literature [35, 36, 38, 40], highlighting the gap between participation rhetoric and practice. This gap illustrates the need for a deeper exploration of participatory barriers and bottlenecks, which motivates this study’s investigative aim.

### Objectives

The emergence of the *Participation Revolution* within humanitarian consciousness alongside an increasingly innovative digital landscape provides a worthwhile juncture to explore how these phenomena intersect; to cooperate or collide [41, 42]. Despite increasing attention, there remains a disconnect between the rhetoric and practice of involving CAP meaningfully [40]. Exploring the *Participation Revolution* from the organisational perspective of current digital health practitioners can reveal the participatory reality in digital health interventions in low-or-middle-income country (LMIC) crisis contexts. Understanding this can highlight areas for improvement and further innovation in an increasingly digitalised landscape. Therefore, this study aims to explore participatory action, as outlined in the LPMF qualitative indicators [37], within contemporary humanitarian digital health projects in LMIC crises. The specific objectives are to: (a) investigate how participatory action manifests within contemporary humanitarian digital health projects, according to the perspectives of humanitarian health practitioners, and (b) within these findings, to explore the breadth and depth to which CAP participate in these projects, and (c), to identify barriers to CAP participation and key lessons in the humanitarian sphere of digital health interventions.

## Methods

### Study design

We conducted online semi-structured key informant (KI) interviews with 16 humanitarian and health practitioners and experts with experience in digital health projects in LMIC crisis contexts. To achieve the objectives of the study, the interview guide (additional materials 1) was structured according to the LPMF qualitative indicators [37] (section six). These are (1) the participation of CAP in humanitarian response [37] and (2) the engagement of CAP in developing humanitarian policy-setting [37]. Ethical approval was applied for and received from the University of Bremen Ethics Office prior to the study’s commencement (reference: 2022-26). This research was guided by the Criteria for Reporting Qualitative Research (COREQ) [43] (additional materials 2).

### Eligibility and sampling

This study sought out current humanitarian and health practitioners with experience in health projects that relied upon digital health tools in LMIC crisis contexts. The eligibility criteria included those people from any country with recent (within the last ten years) experience at any project stage, and in any capacity, of any digital health tool projects where CAP use the tools themselves to address/ prevent/ promote/ manage any health issue in any LMIC with crisis or displacement context. KIs were identified through purposive sampling from internet searches, online professional platforms, and relevant published studies. This was followed by snowball sampling with recommendations and connections from others.

### Recruitment and consent

The team aimed for at least 20 KIs. Over 200 invitations were sent via email or online messaging requesting study participation for themselves or for recommendations of relevant contacts with an accompanying information sheet outlining the purpose and practicalities of the study. The final sample size was 16 due to a lack of willing or eligible participants. Reasons for declining the invitation included a lack of crisis or digital experience or the infancy of the digital health tool. Eligibility was assured through a set of questions to all those who responded positively to the invitation. Following this, a plain language consent form was sent out via email, and all KIs returned a signed copy before data collection commenced. There was no personal or professional relationship between the study team and the KIs.

### Data collection

Data was managed under the General Data Protection Regulation (GDPR) [44]. Interviews with KIs were held between January and June 2023 using online conferencing technology (Zoom and Microsoft Teams) (JB). They lasted between 40 and 90 minutes , were recorded, and then transcribed verbatim. Following this, transcripts were quality checked and anonymised, removing identifying characteristics and replacing them with generic markers. At this point, the original interview recordings were deleted.

### Data analysis

Analysis was both inductive and deductive: Transcribed, anonymised interviews were subjected to a familiarisation process in which the entire dataset was read. The transcripts were then imported into MAXQDA 2020 software and deductively coded according to the LPMF qualitative indicators (JB) and grounding characteristics (JB). Inductive coding and analysis were carried out according to emerging themes elicited from the interview discussions due to the recurring presence or relevance to the investigated topic (JB).

**Fig. 1:**
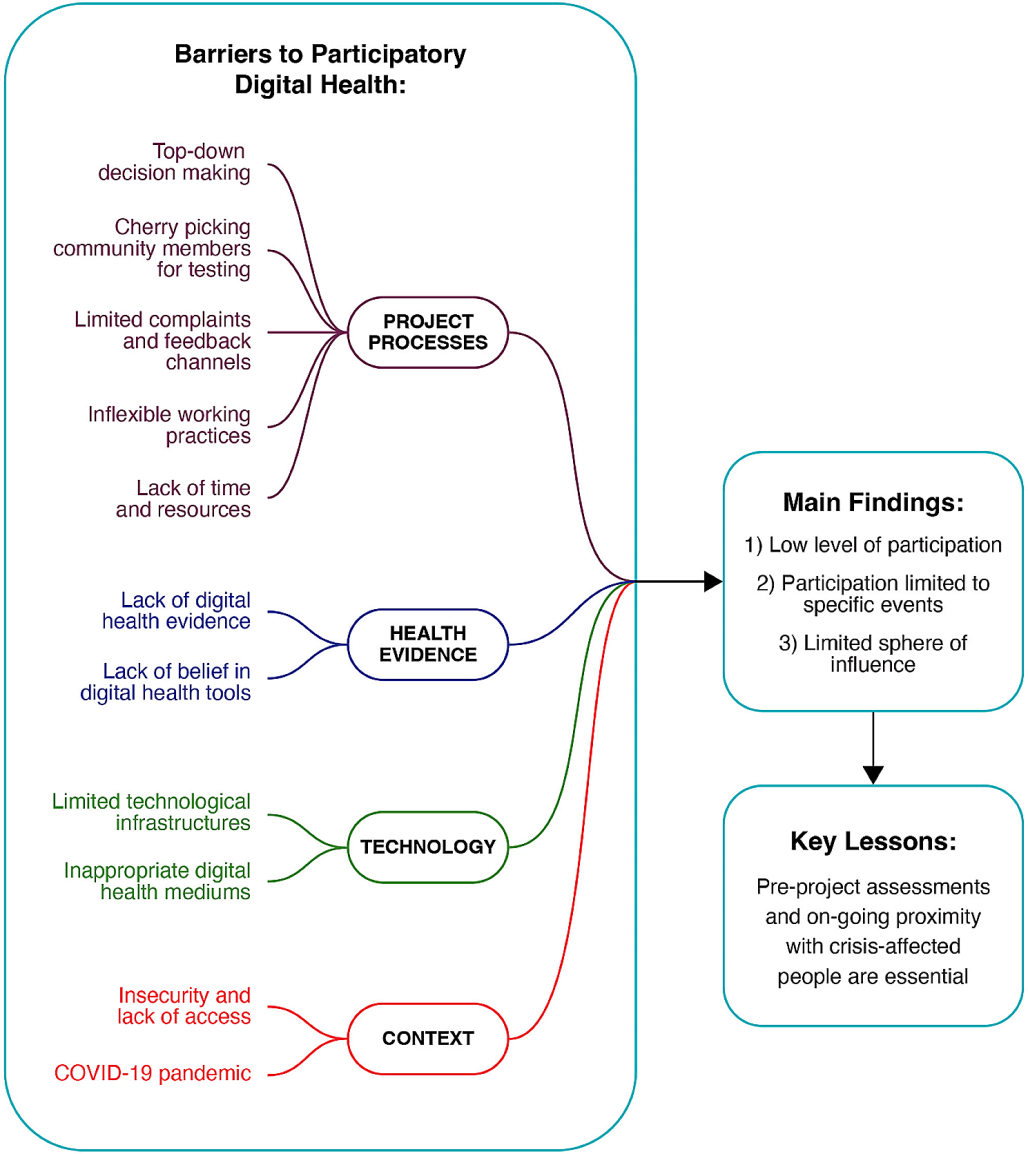
Thematic framework outlining the emerging barriers to greater CAP participation in the humanitarian sphere of digital health interventions, along with main findings and lessons learnt

A second coder (ML) carried out a quality control review of the coding in MAXQDA 2020, adding additional themes and completing any gaps in coding. A consensus was agreed upon between the two reviewers based on the completeness of the coding. From here, the thematic codes were grouped under a parent-child hierarchy (Fig. 1), which developed into the final thematic framework according to the stages of the Framework Method [45].

## Results

### LPMF progress

In relation to the LPMF indicators, KIs reported a limited level of CAP participation and influence in determining the type of humanitarian assistance they received. Generally, KIs stated that CAP were not actively involved in assessing humanitarian needs, prioritising decisions, identifying recipients, deciding upon assistance types or developing humanitarian and health policies and standards.

*“We didn’t involve the actual sample community or population. They had limited influence”* (KI 19).

In order to comprehensively explore the relatively limited LPMF progress further, findings have been structured according to the four major emerging themes. These are weak project processes, lack of health evidence, limited technology, and limiting contextual factors. Within these themes are the individual barriers and bottlenecks relating to greater involvement with CAP in digital health interventions, as identified by the KIs (Fig. 1). To accompany this, the characteristics of the KIs, the crisis contexts and a summary of the digital health tools can be found in additional materials 3.

### Project processes

Rather than participatory planning and collaborative project development, response efforts were reportedly pre-planned and designed by the humanitarian organisations themselves, largely through a top-down approach.

*“I think that [the] management team kind of decides on how we want to, whether we want to start it, whether we want to continue, whether we want to scale it up or shut down”* (KI 17).

However, there were a couple of notable exceptions to this where CAP discussed their daily lives in relation to their health statuses as a method to understand digital behaviours and social challenges to inform project designs.

*“We’re engaging patients in co-creation throughout the entirety of the process*” (KI 11).

Despite a relatively low level of influence reported in general, the degree of participatory power that CAP experienced increased during testing phases, validating, and monitoring digital health prototypes and pilot projects. This mainly took the form of focus group discussions, house-to-house surveys, and interviews. With a few exceptions, participatory engagement was limited to specific project milestones, including monitoring and testing events, and did not extend throughout the project lifecycle or post-intervention.

The selection and eligibility criteria for CAP participation activities were not always clear. Convenience sampling and involving team acquaintances were common methods for prototype testing. Furthermore, sample representativeness in relation to social or crisis dimensions and whether they were incentivised was not well known.

*“Certain community members are cherry-picked to test prototypes on, and [we] incorporate their feedback into the application”* (KI 16).

There were few dedicated mechanisms for capturing and considering CAP feedback or complaints and few systematic approaches for integrating feedback.

*“Complaints were reported ad-hoc… Sometimes these are not reported or not reported properly”* (KI 16).

*“a lot of it is…just based on one person’s power”* (KI 17).

Differences between the digital provider’s working hours and the times that CAP used the digital tools did not always align, which challenged full participatory engagement in provider-led or scheduled services. However, stand-alone digital tools offered greater convenience in their flexibility. One digital health tool was registered as a randomised trial, which hindered it’s flexibility in being adapted according to emerging intervention findings.

The short-term duration of humanitarian projects was identified as a contributing factor to the limited involvement of CAP in participatory activities. The time constraints associated with short timeframe projects and the need for rapid assistance delivery with limited budgets left minimal opportunity for meaningful community engagement and understanding of CAP or the crisis context. Consequently, the focus tended to prioritise technical tool development and testing over comprehensive engagement with and learning from CAP. KIs acknowledged that this resulted in knowledge gaps regarding CAP digital literacy, health literacy, as well as cultural and context information.

*“I think due to time constraints and various other elements at play, a lot of the focus ended up going into the technical development. And maybe not enough attention went to the actual content. The technology is really just a vessel for what you put in it”* (KI 11).

KIs reported that digital health tools were no longer supported despite ongoing health needs when project grants expired due to insufficient health evidence, highlighting an ethical shortcoming where participation or community engagement could have informed decision making.

*“The NGO didn’t really plan for the future of how they would use that. They only want to use something that has been shown to be effective.”* (KI 19).

### Health evidence

In some contexts, the novelty of digital health tools caused a lack of community understanding regarding their benefits, hindering participation and uptake. This was exacerbated by a lack of evidence demonstrating improved health outcomes as a result of using digital health tools.

*“The engagement with available services was a huge issue… some of the study coordinators said [to CAP], “You all said you wanted treatment, we offer it, and you don’t take it, what’s wrong?”* (KI 19).

Likewise, not all providers were convinced regarding the feasibility of digital health tools over more traditional methods. Most evidence bases relied on anecdotal reports without meaningful participation of CAP.

*“A lot of factors go in. And in a vulnerable setting or in a resource-limited setting, it’s very difficult to make that corroboration. So, we kind of rely more on the qualitative aspects”* (KI 17).

This created a disconnect with broader health services and hindered management-level buy-in. Consequently, parallel systems were utilised, resulting in increased or duplicated workloads. Despite this, digital health projects were discussed favourably by KIs for reducing patient waiting times, improving health communications, delivering up-to-date health information, and supporting CAP in health decision-making.

### Technology

The limitations of the technological environment meant that some digital health tools failed or were functionally curtailed by technological infrastructures and therefore did not produce the anticipated outcomes. In turn, this impacted the willingness and interest of CAP to participate in the development and uptake of tools that were not fully functional or reliable within their context.

*A lot of times, a lot of NGOs come with this very sophisticated, very high design innovation prototypes and models. And those aren’t exactly very tech friendly for either implementing partners or for the patients or for the whole ecosystem in general… (KI 17)*.

However, where appropriate digital mediums were operationalised, users appreciated the discretion offered by digital tools in addressing stigmatised health issues over face-to-face services.

*“Because there’s a stigma with accessing mental health services. So, some of them said they don’t really want other people to know about that… It’s [the digital health tool] more discreet*” (KI 19).

### Context

During the COVID-19 pandemic, participatory activities with CAP for the purpose of learning or monitoring and evaluation were largely halted. Similarly, some traditional healthcare routes were put on hold or closed completely in line with restrictions. Whilst this reduced in-person healthcare access in general, it motivated a greater drive towards a digitalised healthcare service provision.

*“One of the best outcomes is that all the [targeted digital health users] in [the digital health programme] got significantly more sessions [than those in traditional treatment programmes]. Because they could get them even when the roads were closed”* (KI 19).

However, participatory events with CAP were not digitalised in the same way. Instead, these were generally deprioritised and did not receive the same attention. This resulted in both participation and response gaps for harder-to-reach communities and groups without digital access.

### Key informants’ lesson learned for leveraging successful digital health interventions

There were two key participatory lessons reported by KIs following their digital health experiences that were reported as enabling factors for successful digital health interventions. The first lesson discussed the importance of understanding CAP and the crisis context from the beginning to inform health projects.

*“I was just kicking myself that I should have done some qualitative evaluation to understand more the dynamics”* (KI 1).

*“My first strategy would not be to just jump in [to the crisis context] and just implement it. I would really want to engage before I actually want to build on it. And that’s not usually how a lot of international organisations will do. They just think they have a good idea. And so, it needs to be implemented everywhere they go. And that’s like the worst thing that you can do in this kind of a situation. They create more damage than good in such situations”* (KI 17).

The benefits of initial assessments were reported.

*“if you do a very thorough in-depth assessment before you actually implement, chances are that it would succeed. You need to have an understanding of the context where you’ll be working. You need to have an understanding of the demographics that you’re working with, the healthcare providers that you will be supporting …all of those are aspects that can really help in moulding the way that you want to build your project”* (KI 17).

The second key lesson reported was that working in proximity to CAP throughout the whole project cycle is the preferred method due to the ongoing contextually and culturally beneficial information that could be gained from these interactions.

*“You need to keep them engaged as much as possible…because I think they’re the ones who kind of understand what the needs are and understand how you can contextualise it or adapt it as much as possible”* (KI 17).

## Discussion

### Progress towards the participation indicators

The LPMF sets forth the aspiration of achieving a *“fuller and more influential involvement of affected people in what relief is provided to them, and how”* [37]. According to the framework, this can be accomplished by ensuring that CAP actively shape and participate in humanitarian response initiatives [37]. . The findings of this study indicate that despite digital health practitioners having a positive perception of participatory action and the benefits it can yield, participatory events were primarily limited to specific, pre-defined project milestones rather than spanning the entire project lifecycle or at the pre- or post-intervention points. In general, participation within the humanitarian digital health sphere manifested as focus groups, surveys, and interviews, and included assessments and testing. CAP influence within these events was curtailed and limited according to organisational boundaries. For the most part, CAP were not actively engaged in needs assessments, prioritisation decisions, or shaping humanitarian and health policies. As a result, CAP held limited influence or power over problem identification, goal definitions, strategy selection or policy shaping. However, notable exceptions were identified, where co-creation activities were enacted with CAP to gain insight into broader lifestyle and digital realities to inform project planning and decisions prior to design phases. , Despite these noteworthy instances, in the context of the LPMF, this study found the overall progress towards participation indicators remains far from a revolution.

### Summary of emerging themes

Participatory barriers emerged as major themes: Project processes, technological limitations, contextual issues, and a lack of health evidence all influenced the participatory reality of digital health projects in LMIC crisis contexts. Key participatory lessons for successful digital health interventions were acknowledging the importance of and acting on in-depth pre-intervention assessments, in conjunction with maintaining ongoing proximity and meaningful engagement with CAP during and after interventions to understand the intervention against evolving humanitarian needs. Our findings indicate that substantial disparities persist between these participatory best practices and the realities of digital health humanitarian action [46, 47].

### Systemic issues

Many of our thematic findings stem from systemic issues. This is symptomatic of the challenges with the current response ecosystem [16, 17] and, thus, distracts us from the human dimension that should be at its core. As a result, incorporating the fundamental right of CAP to participate in decisions that affect them meaningfully has become deprioritised. Consequently, this study emphasises the necessity to move away from a systemic master, and instead towards a person-centred approach. Enhancing collaboration between humanitarian health actors and CAP could surmount identified obstacles and harness the potential offered by digital health tools in addressing humanitarian crises. As an alternative focus, integrating the participation of CAP through the key benchmarks laid out in the Core Humanitarian Standard [48] unlocks a number of ethical gains, such as respect for cultural and contextual norms, equitable representation and diversity inclusion, as well as accountability, non-maleficence, beneficence and mutual reciprocity, informed consent and decision-making powers.

### Overcoming participatory barriers

Barriers to CAP participation caused by project processes include limited time and resources, inflexible working practices, top-down decision-making, and cherry-picking community involvement. Drawing upon the experiences documented by Fitz-Gerald and others [16, 17], our study underscores the persistent challenges of the paternalistic humanitarian system acting in its own interests and restricting the quality and quantity of participatory events [49]. This highlights the tensions between the dominant system approach, and how neglecting contextual and human dimensions undermines adherence to humanitarian principles of humanity, impartiality, neutrality, and independence [50] and impedes progress towards meaningful transformation.

Our research echoes the concerns raised by Ehrenzeller [51] and others [52–54] regarding the intersectionality of localisation required to effect change. To overcome these barriers, digital health projects should embrace all dimensions of the *Localisation Agenda*, centred by the *Participation Revolution*, including decentralising funding, visibility, relationship and capacity building [53]. Enacting these dimensions simultaneously can support the development of a more human-centric approach at all stages and overcome existing constraints.

A lack of digital health evidence posed a barrier for both digital health providers and CAP. This limitation led providers to establish parallel data collection and reporting systems. In turn, this was said to be responsible for community scepticism and lack of trust rather than acceptance of and participation in digital health tool interventions. This contrasts with findings from other feasibility studies [55–57] that showed positive community assessments of digital health tools, highlighting the need for further exploration.

To address this lack of health evidence, digital health projects could actively engage healthcare providers and CAP in recognising the potential impacts of digital tools on health outcomes. Implementing co-production initiatives presents an opportunity for this and can foster interest and trust by reporting outcomes and interpreting results collaboratively. This approach can promote a learning culture that strengthens support for digital health initiatives and ensures their alignment with real-world challenges.

In settings with considerable technological limitations, as observed in several studies [35, 58], barriers such as poor connectivity pose a considerable challenge to both digital health interventions and digital participatory action. When technology and infrastructures do align, digital health tools become powerful facilitators in addressing specific health issues, particularly for stigmatised problems such as mental health, offering privacy and discretion [59]. This represents a considerable strength for digital health tools, considering the high burden of mental health issues found within crisis contexts [60, 61].

However, as our exploration highlights, the reality of aligning tools within limited techno-ecosystems is not always feasible and underscores the importance of designing interventions according to local contexts. Incompatible digital tools that are not tailored to the hosting environment are indicative of hierarchical provider-led decision-making rather than user-led approaches prioritising the needs and desires of CAP [62, 63]. As WHO outlines, digital tools are only a platform, not a means to an end [42]. A greater understanding of existing digital behaviours in relation to socio-economic aspects, such as gender, ownership, and literacy can inform appropriate digital platform selections and local hosting capabilities [61]. An important consideration here is that certain contexts may not be viable for digital health tools, and traditional methods may be more appropriate. This decision should be locally led.

As outlined by other studies [64, 65], in regions with ongoing conflicts, accessibility challenges between responders and CAP can hinder access to services [66] as well as traditional participatory activities [67–69]. In such contexts, greater community engagement and improved participation can provide protection and access to humanitarian responders. This must go hand-in-hand with principled action but can act as a mechanism in which assistance can continue to be delivered to communities within these areas.

However, given the shrinking humanitarian space, increasing human rights violations, and attacks on responders [67], proximity with CAP may not always be possible. In these cases, digitalised health services offer the potential to transcend some of these barriers virtually, as demonstrated during the emergence of the COVID-19 pandemic. The pandemic served as a catalyst for digitalising health and humanitarian sector services, and as a result, digital interventions may have extended the reach of health services and fostered greater inclusion of CAP in humanitarian response efforts [70]. This is a notable achievement and may become increasingly important in the future. Establishing best practices and effective digital health platforms that also incorporate digitalised participatory engagement opportunities would be highly advantageous in this evolving landscape.

It is crucial to acknowledge that while increased digitalisation may have broadened inclusion for some communities in accessing services, it risks excluding others [71, 72]. As outlined by Boza-Kiss et al. [73], the lack of access to digital technology in low-income crisis contexts disproportionately affects socio-economically disadvantaged communities. Consequently, balancing the benefits of digital health solutions with addressing disparities is crucial to ensure equitable and effective support for all CAP during crises.

### Implications

Insufficient CAP participation in digital health projects has several critical implications. Firstly, poor participation with CAP can result in unrepresentative local information lacking cultural and contextual nuances, limiting project design and development. Secondly, the lack of engagement may limit the targeted users’ adoption of digital health tools. Thirdly, without the meaningful involvement of CAP, digital health tools may not be fit for purpose, technologically, economically, ethically, or socially. This may mean that intended groups are not reached with humanitarian response efforts or that ethical lines are crossed, which could result in adverse outcomes.

This study’s findings align with existing literature and best practices in health and humanitarian endeavours [74, 75] in highlighting two critical lessons learned for the success of digital health projects: Firstly, conducting comprehensive population and context assessments *with* CAP before project initiation is crucial for ensuring appropriate design and implementation: Building on the lessons learned from the West African Ebola outbreak [76, 77] and COVID-19 Pandemic [78, 79], we emphasis this as an ethical approach for deeper, more holistic understanding of community needs. In this way, CAP participation can help to reduce power imbalances and systemic paternalism whilst bridging the divide between global resources and local solutions [80].

Secondly, maintaining close proximity and ongoing interaction with CAP throughout and after humanitarian actions is essential [66, 81–83]. Previous studies have shown that it is not enough to simply develop and implement a digital health tool [84]. Instead, ongoing engagement is required to facilitate trust and legitimacy whilst enabling continuous adaptation and improvement, maximising the potential benefits of digital health interventions within evolving and dynamic environments. Incorporating these lessons into digital health projects can uphold a rights-based approach that increases accountability to CAP whilst contributing to more effective and impactful response efforts.

### Strengths and limitations of the study

In seeking to understand the organisational perspective of the *Participation Revolution*, this study engaged with digital health and humanitarian organisations, recognising their pivotal role and access to resources required for an accountable and effective response. As highlighted in much literature, the interest of CAP to participate is high [85], and whilst our study offers a valuable organisational perspective, the limitations of not including CAP in this study are acknowledged. Participatory rights are the cornerstone for the realisation of numerous fundamental ethical and human rights, including, but not limited to, the right to non-discrimination, the right to healthcare and the right to freedom of expression. Further research in this field should explore this topic from the CAP perspective, exploring CAP’s awareness of their rights in relation to health, digital and humanitarian response efforts. Investigating participatory evaluation mechanisms as indicators of success, measured by CAP, for humanitarian and digital health interventions could contribute towards the evolving discourse on inclusive and rights-based humanitarian and digital health interventions. Furthermore, given the diversity of health issues addressed by technology, additional analysis of these and their crisis contexts to understand their potential participatory opportunities could enhance this research field.

We recognise this study’s small sample size as a limitation for generalisability and as such cannot tease out differences between technology types, health issues, and crisis contexts. However, considering this against the diversity of CAP and crisis contexts globally, we consider this study a baseline for greater exploration in this sphere. Considering the complexity of the topic, this study’s strength lies in the rich diversity of perspectives from several humanitarian crises, crisis context types, types of digital health tools and health issues.

The lack of health evidence from digital health tools within humanitarian contexts challenges the very notion that they benefit health outcomes. Further research to demonstrate their ability to affect positive health change could garner greater traction as well as highlight opportunities for further CAP participation in response efforts.

## Conclusion

This study has highlighted the gap between participatory rhetoric and practice and highlighted the barriers that shape the extent and form of participation within humanitarian digital health response efforts. Despite widely accepted participatory benefits, this study found only a few examples of strong participatory practices. To further the *Participation Revolution* within the digital health humanitarian paradigm, systemic tensions with project processes, contexts, technologies, and health evidence should be addressed to achieve a more inclusive and collaborative humanitarian response. This paper has offered a number of strategies to overcome or minimise participatory challenges and humanitarian digital health interventions could benefit from these through greater promotion and prioritisation of meaningful, powerful participation with CAP across the intersectionality of the Localisation Agenda.

### Electronic supplementary material

Below is the link to the electronic supplementary material.

Supplementary Material 1

Supplementary Material 2

Supplementary Material 3

Supplementary Material 4

## Abbreviations

App: Application (digital)
CAP: Crisis Affected People
COREQ: Criteria for Reporting Qualitative Research
COVID-19: Coronavirus Disease 2019
GDPR: General Data Protection Regulation
IASC: Interagency Standing Committee
IDPs: Internally Displaced Populations
KI: Key Informant(s)
LMIC: Low- and Middle-Income Country/ies
LPMF: Localisation Performance Measurement Framework
NGO: Non-Governmental Organisation
QI: Qualitative Indicator(s)
SMS: Short Message System
SOP: Standard Operating Procedure(s)
