# Supplementary material for: Exploring the perspectives and practices of humanitarian actors towards the Participation Revolution in humanitarian digital health responses: a qualitative study
Source: Global Health. 2024 Apr 26;20:36. doi: 10.1186/s12992-024-01042-y (PMC11055264; doi:10.1186/s12992-024-01042-y)
Supplement: Supplementary file 1 — Supplementary Material 1 [file 12992_2024_1042_MOESM1_ESM.docx]

**Additional Materials 1: Qualitative Semi-Structured In-depth Interviews with Key Informants**

| **#** | **Questions** | **Prompts/probes** |
| --- | --- | --- |
|  |  |  |
| 1 | - In relation to the *digital health tool intervention* can you give me an overview of your role and your work within the humanitarian field? | - Who do you work for? - What is your job? - What does this include? - What is your background? - How long have you done…? - What is your expertise? - Where have you worked? - What are the tools/platforms? - What health issues do they seek to address? - How do they work to impact health? - What are the target groups? Why? - How many people use them? - How successful are they? - What do you think about them? |
| 2 | - Did crisis-affected populations participate in this *digital health* project? - What were the main insights you received from crisis-affected people? - How were these integrated into the project? - How were the participation mechanisms promoted? - Did your organisation have an operational complaints mechanism? | - At what stage(s)? - How & to what extent did they participate/ collaborate? *(FGD? Interviews? Surveys? Feedback mechanisms? Etc.?) 1 way? 2 way? Multiple?* - How did it go? - What could have been done better? - Was it used in this instance? - How successful was it? |
| 3 |  |  |
| 4 | - Within the timeframe and activities of the *digital health project,* what information was provided to targeted crisis-affected populations about your organisation generally? - How was it provided and received? | - Visibility? - Community meetings? - Door to door? - General information on humanitarian principles? Organisational mandates? Funding streams? Etc? |
| 5 | - Within the time and activities of the *digital health project* implementation, who made the project decisions? How were they made? - Was context analysis carried out specifically for *this digital health intervention*? - Were relevant crisis-affected people involved in this activity? | - Where does the power lie? - What kind? By who? Who was involved? - How? To what extent? |
| 6 | - Does your organisation have a community engagement/participation/ localisation or similar policy or framework that guides interaction with crisis-affected people? - Were crisis-affected people involved in standards setting? | - Was it used in this instance? - How successful was it? - Who was involved in the development of this? |
| 7 | - What are the impacts of using this *digital health tool**? | - Are health outcomes of users being improved? How do you know this? What data is being collected? By who? How often? What does it tell you? - Do you believe the participation of relevant crisis-affected people have contributed to this? Why? - What else could have been done here? |
| 8 | - If you could do this work all over again, what would you change and what would you keep the same? | - Lessons learned? - strengths, weaknesses, opportunities and threats? |
| 9 | - Is there anything I haven’t asked you that you think is important to say in this interview? | - What? Why? How? Who? Where? When? |
