# Supplementary material for: Exploring the perspectives and practices of humanitarian actors towards the Participation Revolution in humanitarian digital health responses: a qualitative study
Source: Global Health. 2024 Apr 26;20:36. doi: 10.1186/s12992-024-01042-y (PMC11055264; doi:10.1186/s12992-024-01042-y)
Supplement: Supplementary file 2 — Supplementary Material 2 [file 12992_2024_1042_MOESM2_ESM.docx]

**Additional Materials 2: COREQ Statement**

Study title: Exploring the Perspectives and Practices of Humanitarian Actors Towards the Participation Revolution in Digital Health Humanitarian Responses: A Qualitative Study

| **Topic** | | **Item No.** | **Guide Questions** | **Responses** | **Page number** | |  |
| --- | --- | --- | --- | --- | --- | --- | --- |
| Domain 1: Research team and reflexivity | | | | | |  | |
| *Personal Characteristics* | Interviewer/ facilitator | 1 | Which author/s conducted the interview or focus group? | Jennifer Benson | Data analysis section, page 9 | |  |
|  | Credentials | 2 | What were the researcher’s credentials? | Master of Science, Global Health | Reflexivity statement – additional materials 4 | |  |
|  | Occupation | 3 | What was their occupation at the time of the study? | PhD Research Candidate |  |  |  |
|  | Gender | 4 | Was the researcher male or female? | Female |  |  |  |
|  | Experience & training | 5 | What experience or training did the researcher have? | Extensive experience in humanitarian responses in LMIC contexts. 3 years of experience conducting qualitative research |  |  |  |
| *Relationship with participants* | Relationships established | 6 | Was a relationship established prior to study commencement? | No | Recruitment & consent section, page 8 | |  |
|  | Participant knowledge of the interviewer | 7 | What did the participants know about the researcher? e.g., personal  goals, reasons for doing the research | The researcher’s goals were to complete the objectives of the research and explicitly in the study invitation and orally at the beginning of the interview. There was no personal gain in the completion of research activities. |  |  |  |
|  | Interview characteristics | 8 | What characteristics were reported about the interviewer/ facilitator? e.g., Bias, assumptions, reasons and interests in the research topic | The interviewer introduced herself as working in a health research institute in Germany and completing this research as part of her PhD work. She disclosed a background in humanitarian response work. | Reflexivity statement – additional materials 4 | |  |
| Domain 2: Study Design | | | | | |  | |
| *Theoretical framework* | Methodological orientation  and Theory | 9 | What methodological orientation was stated to underpin the study? e.g.  grounded theory, discourse analysis, ethnography, phenomenology,  content analysis | A semi-structured interview approach is outlined. This was structured in accordance with the Localisation Performance Measurement Framework indicators within section 6 – Participation Revolution. | Methods section - page 8 | |  |
| *Participant selection* | Sampling | 10 | How were participants selected? e.g. purposive, convenience, consecutive, snowball | Purposive sampling was used to select participants. Snowball sampling followed this in an attempt to capture maximum variation and representativeness of perspectives and experiences. | Eligibility & sampling section and Recruitment & consent section, page 8 | |  |
|  | Method of approach | 11 | How were participants approached? e.g., face-to-face, telephone, mail,  email | Participants were approached via email or message service within professional network portals with an invitation to participate in the study. They were provided with a study information sheet, containing the study purpose, scope, publishing intentions and data management strategies. |  |  |  |
|  | Sample size | 12 | How many participants were in the study? | We recruited 16 participants. |  |  |  |
|  | Non-participation | 13 | How many people refused to participate or dropped out? Reasons? | 22 participants registered for the study. 6 were lost: 1 declined for eligibility reasons, 1 was no longer available, 4 failed to respond to 3 communications after registering for reasons unknown. |  |  |  |
| *Setting* | Setting of data collection | 14 | Where was the data collected? e.g., home, clinic, workplace | Interviews were held online. Some participants attended from home offices; others attended from their places of work | Data collection section, pages 9 | |  |
|  | Presence of non-participants | 15 | Was anyone else present besides the participants and researchers? | No | N/A | |  |
|  | Description of sample | 16 | What are the important characteristics of the sample? e.g. demographic data, date | Table 1 - 3 outlines the participant’s organisation and their position in relation to the crisis, crisis context types, digital tool types, digital tool user types and the health issues addressed within the digital health tools | Eligibility & sampling section – page 8 and Results characteristic - additional materials 3 | |  |
| *Data Collection* | Interview guide | 17 | Were questions, prompts and guides provided by the authors? Was it pilot tested? | A semi-structured interview guide was used for all interviews. The guide was used flexibly to be most relevant to the diversity of projects and the hybridity of working practices. This included several prompts to probe into certain areas where applicable. This enabled space for participants to raise topics they felt were important and space for exploration of emerging themes. It was not pilot-tested. | Additional materials 1 - Interview topic guide | |  |
|  | Repeat interviews | 18 | Were repeat interviews carried out? If yes, how many? | No repeat interviews were conducted | N/A | |  |
|  | Audio/visual recording | 19 | Did the research use audio or visual recording to collect the data? | Audio and video recording was used for all but one interview (using Zoom). In this case, only audio recording (using Teams) was carried out due to technological capacities | Data collection section – page 9 | |  |
|  | Field notes | 20 | Were field notes made during and/or after the interview or focus group? | No | N/A | |  |
|  | Duration | 21 | What was the duration of the interviews or focus groups? | Between 40 and 120 minutes | Data collection section – page 9 | |  |
|  | Data saturation | 22 | Was data saturation discussed? | It was discussed in team meetings. However, challenges in recruiting eligible participants persisted, and saturation was not met | N/A | |  |
|  | Transcripts returned | 23 | Were transcripts returned to participants for comment and/or correction? | No, this was decided against so as not to further burden participants | N/A | |  |
| Domain 3: analysis and findings | | | | | | |  |
| *Data Analysis* | Number of data coders | 24 | How many data coders coded the data? | Data was coded by the lead interviewer (JB). A coding check was conducted by ML, with the objective of code completion and not coding consensus. | Data analysis section, page 8-9 | |  |
|  | Description of the coding tree | 25 | Did authors provide a description of the coding tree? | Yes, the coding tree was developed from the PR qualitative indicator themes as well as emerging themes resulting from these discussions (figure 1) | Thematic framework figure - page 10 | |  |
|  | Derivation of themes | 26 | Were themes identified in advance or derived from the data? | PR themes were deductively outlined. Emerging themes were inductively identified | Data analysis section, page 9 | |  |
|  | Software | 27 | What software, if applicable, was used to manage the data? | Microsoft Excel for initial data charting and MAXQDA 2020 for data coding |  |  |  |
|  | Participant checking | 28 | Did participants provide feedback on the findings? | No, this was decided against so as not to further burden the participants | N/A | |  |
| *Reporting* | Quotations presented | 29 | Were participant quotations presented to illustrate the themes/findings?  Was each quotation identified? e.g., participant number | Yes, key quotes were presented within the results and discussion sections to illustrate findings and interpretation of these. KIs were each allocated a unique number and these were used following the quotes | Results section – page 10> | |  |
|  | Data and findings consistent | 30 | Was there consistency between the data presented and the findings? | Yes, this was confirmed by two members of the research team (JB & ML) | Data analysis section, page 9 | |  |
|  | Clarity of main themes | 31 | Were major themes clearly presented in the findings? | Yes, these were outlined in summary form and discussed in further detail | Results section – page 10> | |  |
|  | Clarity of minor themes | 32 | Is there a description of diverse cases or a discussion of minor themes? | Yes, these were outlined in summary form and discussed in further detail as lessons learnt | Results section – page 10> & Discussion section – page 16> | |  |

**References:**

Developed from: Tong A, Sainsbury P, Craig J. Consolidated criteria for reporting qualitative research (COREQ): a 32-item checklist for interviews and focus groups. International Journal for Quality in Health Care. 2007. Volume 19, Number 6: pp. 349 – 357
