# Supplementary material for: Exploring the perspectives and practices of humanitarian actors towards the Participation Revolution in humanitarian digital health responses: a qualitative study
Source: Global Health. 2024 Apr 26;20:36. doi: 10.1186/s12992-024-01042-y (PMC11055264; doi:10.1186/s12992-024-01042-y)
Supplement: Supplementary file 3 — Supplementary Material 3 [file 12992_2024_1042_MOESM3_ESM.docx]

**Additional Materials 3: Results characteristics chart**

| **Gender of Key Informant** | **Organisation Type of Key Informant** | **Technology Type of Digital Health Tool** | **Technology Format of Digital Health Tool** | **Health Issue(s) Addressed within Digital Health Tool** | **Digital Health Approach** | **Intervention Type** | **Geographic Region Of Digital Health Deployment** | **Crisis- Affected Persons Type (users)** | **Crisis type (context)** |
| --- | --- | --- | --- | --- | --- | --- | --- | --- | --- |
| Female | International non-governmental organisation  Internationally positioned | Computer/ Smart Phone | Social Media | Health promotion & disease prevention (emergency preparedness, HIV, sexual violence, COVID-19, mental health) | At-home advice & health services signposted | Standalone tool | Africa, Asia, Middle East, Caribbean, Latin America | Displaced people, refugees, outbreak-affected | Conflict, outbreak |
| Male | International non-governmental organisation  Internationally positioned | Phone/ Smart Phone | Telemedicine Consultation | Mental health | Psychological first aid therapy | Connected to broader health intervention | Asia, Middle East | Displaced people, minority populations | Conflict, impoverished areas, urban slums |
|  |  | Phone/ Smart Phone | Telemedicine Consultation | Non-communicable & communicable diseases (diabetes, Tuberculosis) | Management, support & therapy | Connected to broader health intervention | Asia, Middle East | Displaced people, minority populations | Conflict, impoverished areas, urban slums |
| Female | International non-governmental organisation  Crisis-affected KI | Computer/ Mobile Phone | Social Media | Health promotion & disease prevention (health care services promotion, including for surgical care, emergency preparedness, mental health) | At-home advice & health services signposted | Standalone tool | Middle East | Displaced people | Conflict, outbreak |
| Male | National non-governmental organisation  National KI to crisis | Smart Phone | Smart Phone Application | Vaccination health promotion | Appointment reminders, health records, health services signposted | Standalone tool | Asia | Refugees | Conflict |
| Male | International non-governmental organisation  Internationally positioned | Smart Phone | Smart Phone Application | Palliative care | Pain level diagnoses and management | Connected to broader health intervention | Africa | Refugees | Conflict |
| Male | International non-governmental organisation  Crisis-affected KI | Website/ Mobile Phone | Social Media | Health promotion & disease prevention (poison prevention, COVID-19, antibiotic resistance) | At-home advice & health services signposted | Standalone tool | Middle East | Displaced | Conflict |
| Male | International non-governmental organisation  Internationally positioned | Smart Phone | Smart Phone Application | Disease outbreaks | Outbreak surveillance & tracking | Connected to broader health intervention | Asia | Displaced | Conflict |
| Female | United Nations Agency  National KI to crisis | Internet Enabled Device | Shared Drive | Health promotion & disease prevention (Water sanitation and hygiene) | Health services signposted | Standalone tool | Asia | Displaced | Disaster/ climate-related emergency |
| Female | International non-governmental organisation  Internationally positioned | Phone/ Smart Phone | Telemedicine Consultation | Non-communicable & communicable diseases (Diabetes, breast cancer screening support) | Consultations with healthcare professionals | Connected to broader health intervention | Africa, Asia, Middle East, Latin America | Displaced, refugees, outbreak affected | Conflict, Disaster/ climate-related emergency |
| Male | Academic organisation  Internationally positioned | Smart Phone | Smart Phone Application | Vaccination health promotion | Appointment reminders, health records, health services signposted | Connected to broader health intervention | Middle East | Refugees | Conflict |
|  |  | Mobile Phone | Short Message System (SMS) | Vaccination health promotion | Appointment reminders, health services signposted | Connected to broader health intervention | Africa | Displaced | Conflict |
|  |  | Mobile Phone | Short Message System (SMS) | Vaccination health promotion | Appointment reminders, health services signposted | Connected to broader health intervention | Africa | Displaced | Conflict |
|  |  | Mobile Phone | Short Message System (SMS) | Disease outbreaks | Outbreak surveillance & tracking | Connected to broader health intervention | Africa | Displaced | Conflict |
| Male | Academic organisation  Internationally positioned | Mobile Phone | Short Message System (SMS) | Communicable diseases (Scabies, diarrhea) | Screening & at-Home Treatment Plans | Standalone tool | Asia | Displaced | Disaster/climate-related emergency poverty-stricken |
|  |  | Mobile Phone | Short Message System (SMS) | Non-communicable diseases (diabetes) | Screening & treatment plans | Standalone tool | Asia | Displaced | Disaster/ climate-related emergency |
|  |  | Smart Phone | Smart Phone Application | Mental health | Screening & treatment plans | Connected to broader health intervention | Asia | Refugees | Conflict |
|  |  | Smart Phone | Smart Phone Application | Mental health | Screening & treatment plans | Connected to broader health intervention | Middle East | Refugees | Conflict |
|  |  | Smart Phone | Smart Phone Application | Non-communicable diseases (cancer screening support, high blood pressure) | Screening & health services signposted | Connected to broader health intervention | Asia | Refugees | Conflict |
| Female | International non-governmental organisation  Internationally positioned | Smart Phone | Smart Phone Application | Non-communicable diseases & communicable diseases (Diabetes, Tuberculosis) | At-home treatment plans & communications with healthcare workers | Connected to broader health intervention | Africa, Asia, Middle East, Caribbean, Latin America | Displaced people, refugees, outbreak-affected | Conflict, disaster/ climate related emergency |
| Male | Academic organisation  Internationally positioned | Phone/ Smart Phone | Telemedicine Consultation | Mental health | Telemedicine therapy | Connected to broader health intervention | Middle East | Refugees | Conflict |
| Male | International non-governmental organisation  Internationally positioned | Internet Enabled Device | Telemedicine Consultation | Communicable diseases including managing HIV and tuberculosis | Consultations with healthcare professionals | Connected to broader health intervention | Africa, Asia, Middle East, Caribbean, Latin America | Displaced people, refugees, outbreak-affected | Conflict, disaster/ climate-related emergency |
| Male | Academic organisation  Internationally positioned | Computer/ Smart Phone | Smart Phone Application/ Website | Mental health | Screening & at-home treatment | Standalone tool | Middle East | Refugees | Conflict |
| Male | United Nations Agency  Internationally positioned | Computer/ Smart Phone | Smart Phone Application/ Website | Mental health | Screening & at-home treatment | Standalone tool | Africa, Asia | Refugees | Conflict |

**Summary of results characteristics**

**Table 1**: Key informant characteristics, crisis types and regions

| **Key Informants** | **n=16** |
| --- | --- |
| Male | 11 |
| Female | 5 |
| **Key Informant Organisation Type** | **n=16** |
| International NGO | 9 |
| Academic/Research Organisation | 4 |
| National/Regional NGO | 1 |
| United Nations Agency | 2 |
| **Key Informant Position in Relation to the Crisis *** | **n= 18** |
| Internationally positioned | 12 |
| Nationally positioned | 2 |
| Locally positioned | 2 |
| Crisis-affected themselves | 2 |
| **Crisis Regions*** | **n= 41** |
| Asia | 14 |
| Middle East | 12 |
| Africa | 9 |
| Latin America | 3 |
| Caribbean | 3 |
| **Crisis Type*** | **n= 32** |
| Conflict | 21 |
| Disaster/Climate Related | 6 |
| Poverty-stricken/exclusion | 3 |
| Outbreaks | 2 |

*Some KIs reported more than one position, crisis type and/or crisis region.

**Table 2:** Digital health tool characteristics

| **Digital health tool target users*** | **n= 34** |
| --- | --- |
| Internally displaced (IDPs) | 15 |
| Internationally displaced (Refugees) | 13 |
| Outbreak affected | 4 |
| Minority groups | 2 |
| **Digital health tool technology type*** | **n=33** |
| Smartphones | 15 |
| Mobile phones (feature or basic) | 7 |
| Computer | 5 |
| Phone | 4 |
| Internet-enabled device | 2 |
| **Digital health tool technology format*** | **n=26** |
| Application (app) | 10 |
| Short messaging system (SMS) | 5 |
| Telemedicine (telephone calls) | 5 |
| Social media | 3 |
| Website | 2 |
| Shared Drive | 1 |
| **Stand-alone or connected healthcare intervention*** | **n=24** |
| Complementary healthcare | 15 |
| Stand-alone healthcare | 9 |

*Some KIs provided multiple answers in relation to these characteristics

**Table 3:** Summary of health issues addressed by digital health tools discussed

| **Health issues addressed digitally*** | **n= 24** |
| --- | --- |
| Mental Health | 6 |
| Non-communicable Diseases | 5 |
| Communicable Diseases | 4 |
| Vaccine Promotion | 4 |
| Outbreaks | 2 |
| Water, Sanitation, Hygiene | 1 |
| Poison prevention and care | 1 |
| Palliative care | 1 |

*Some KIs mentioned more than one digital health project or tool or digital health tools that addressed more than one health issue.
