# Supplementary material for: Exploring the perspectives and practices of humanitarian actors towards the Participation Revolution in humanitarian digital health responses: a qualitative study
Source: Global Health. 2024 Apr 26;20:36. doi: 10.1186/s12992-024-01042-y (PMC11055264; doi:10.1186/s12992-024-01042-y)
Supplement: Supplementary file 4 — Supplementary Material 4 [file 12992_2024_1042_MOESM4_ESM.docx]

**Additional Materials 4: Research team and reflexivity**

*Authors:*

Jennifer Benson [benson@leibniz-bips.de](mailto:benson@leibniz-bips.de) ^(1, 2, 3)^

Tilman Brand [brand@leibniz-bips.de](mailto:brand@leibniz-bips.de) ^(2)^

Meret Lakeberg [lakeberg@leibniz-bips.de](mailto:lakeberg@leibniz-bips.de) ^(1, 2)^

*Organisational Affiliations:*

1. Health Sciences Bremen, University of Bremen, Germany
2. Leibniz Institute for Prevention Research and Epidemiology – BIPS, Department Prevention and Evaluation, Bremen, Germany
3. Leibniz Science Campus Digital Public Health, Bremen, Germany

| *Characteristics* | *Description* |
| --- | --- |
| Credentials | All authors have academic degrees: JB holds Master of Science degrees, ML holds a Bachelor of Arts degree, and TB holds a doctoral degree. JB is a doctoral candidate. |
| Occupation | All authors are employed at the Leibniz Institute for Prevention Research and Epidemiology (BIPS). JB and TB are researchers. ML is a student assistant. |
| Age, race, gender, socio-economic status & language skills | The research team consisted of two female and 1 male cis-gendered authors, aged between 20 and 49 years (mean age bracket 40-49). All identified as white, and non-disabled with either high or middle socioeconomic statuses. Collectively they hold British and German citizenship and speak English, German, French and some Portuguese and Dutch. |
| Relevant professional experiences | Between them, the research team have experience in co-authoring papers on quantitative and qualitative research, articles and reviews in health sciences and social sciences. The majority of these have occurred in Germany and the UK. |
| LMIC experiences | The research team has a broad portfolio of relevant experiences of living and working in LMIC [Tanzania, Ethiopia, South Sudan, Iraq, Syria, Gaza, Lebanon, Pakistan & Bangladesh]. These experiences include professional humanitarian health response activities in emergencies as well as cooperation with LMIC scientific colleagues including personnel exchanges, workshops, and joint publications in the field of public health research. Additionally, the team brings experience in supporting refugees with social services within Europe. |
